# Supplementary material for: High Photoresponse Black Phosphorus TFTs Capping with Transparent Hexagonal Boron Nitride
Source: Membranes (Basel). 2021 Dec 1;11(12):952. doi: 10.3390/membranes11120952 (PMC8705758; doi:10.3390/membranes11120952)
Supplement: Supplementary file 1 [file membranes-11-00952-s001.zip › membranes-1461270-supplementary.pdf]

Supplementary Materials for

High Photo Response Black Phosphorus TFTs Capping with Transparent Hexagonal Boron Nitride

Dewu Yue[1,2], Ximing Rong[1], Shun Han[1], Peijiang Cao[1], Yuxiang Zeng[1], Wangying Xu[1], Ming Fang[1], Wenjun Liu[1], Deliang Zhu[1], and Youming Lu[1,2*]

[1] College of Materials Science and Engineering, Shenzhen Key Laboratory of Special Functional Materials, Shenzhen Engineering Laboratory for Advanced Technology of Ceramics, Guangdong Research Center for Interfacial Engineering of Functional Materials, Shenzhen University, Shenzhen 518060, China

[2] Key Laboratory of Optoelectronic Devices and Systems of Ministry of Education and Guangdong Province, College of Optoelectronic Engineering, Shenzhen University, Shenzhen518060, China

[*]Corresponding Author: E-mail: ymlu@szu.edu.cn

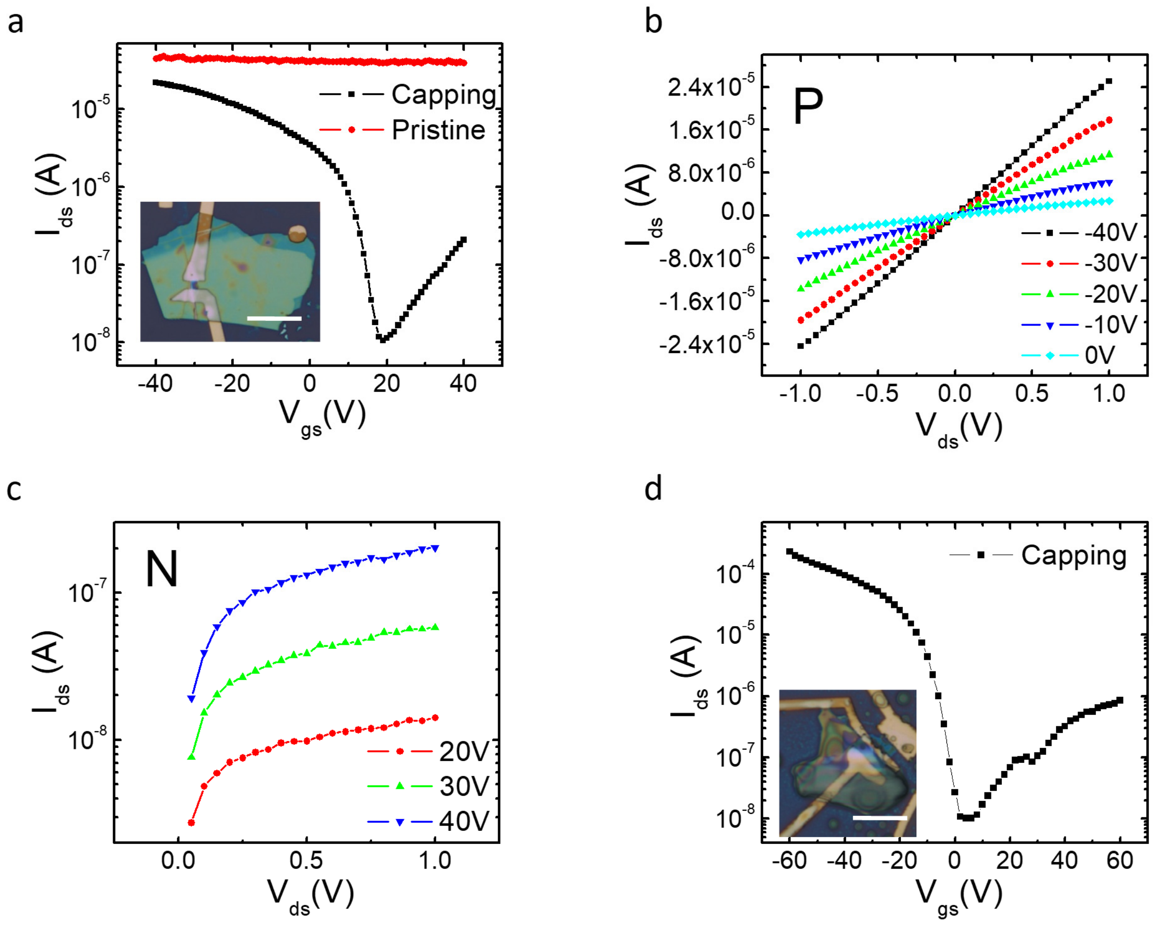

Figure S1, a, shows ambipolar characteristic of h-BN capped BP devices (hBN2) and **b, c,** show output characteristics of the device (hBN2) in P- and N-type operations, respectively. **d,** shows ambipolar characteristic of h-BN capped BP devices (hBN3). The scale bar indicates 5 μm.

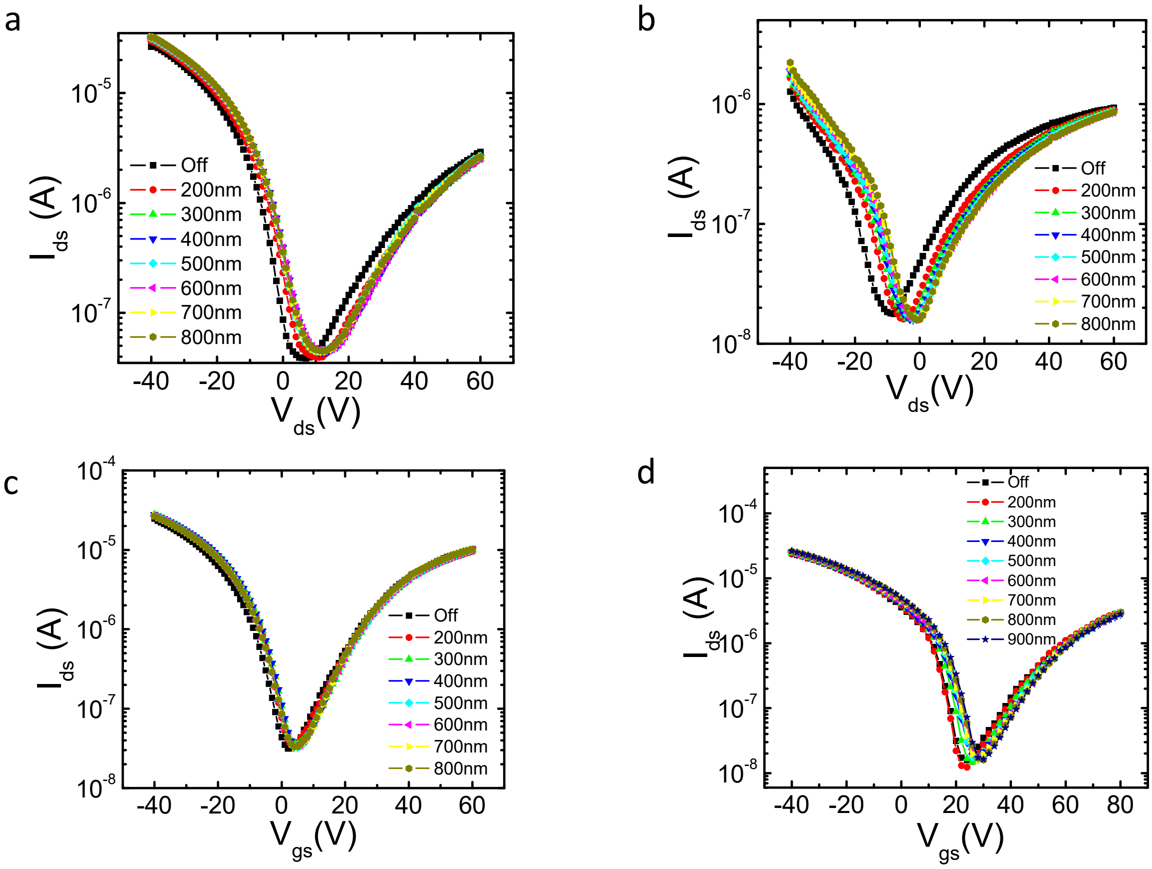

Figure S2 a, b, c, and **d** shows photocurrent generated upon monochromatic light illumination (wavelength ranging from 200 to 900 nm) of BP devices hBN1 and hBN2.

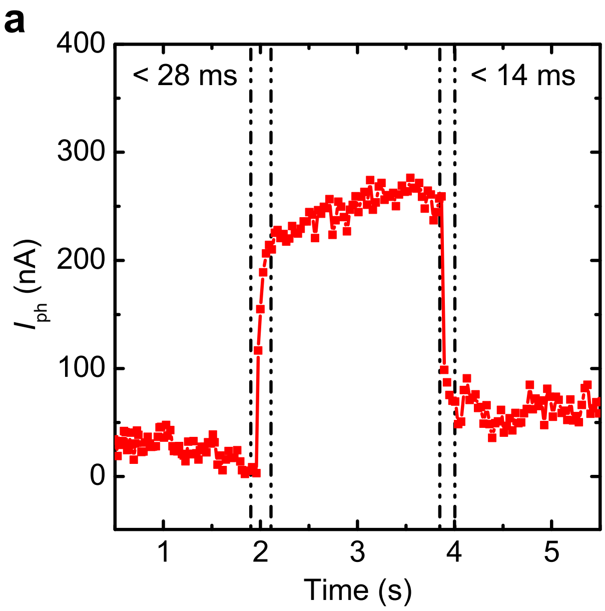

Figure S3, a, The photocurrent response.

**Strategy**	**Electron Mobility (cm^2/V s)**	**Reference**
h-BN capping	78	This work
Al*Ox* encapsulated	<30	Ref.[1]
MoO_3 or Cs_2CO_3 doping	27	Ref.[2]
Ni or Pd contact	<20	Ref.[3]
Al_2O_3 capping	38	Ref.[4]
Gr electrode	40	Ref.[5]

Table S1 Comparision of electron mobility.

References:

1. Wood, J. D.; Wells, S. A.; Jariwala, D.; Chen, K. S.; Cho, E.; Sangwan, V. K.; Liu, X. L.; Lauhon, L. J.; Marks, T. J.; Hersam, M. C., Effective Passivation of Exfoliated Black Phosphorus Transistors against Ambient Degradation. *Nano Lett.* **2014,** *14* (12), 6964-6970.
2. Xiang, D.; Han, C.; Wu, J.; Zhong, S.; Liu, Y. Y.; Lin, J. D.; Zhang, X. A.; Hu, W. P.; Ozyilmaz, B.; Neto, A. H. C.; Wee, A. T. S.; Chen, W., Surface transfer doping induced effective modulation on ambipolar characteristics of few-layer black phosphorus. *Nature Communications* **2015,** *6*.
3. Du, Y. C.; Liu, H.; Deng, Y. X.; Ye, P. D., Device Perspective for Black Phosphorus Field-Effect Transistors: Contact Resistance, Ambipolar Behavior, and Scaling. *ACS Nano* **2014,** *8* (10), 10035-10042.
4. Liu, H.; Neal, A. T.; Si, M. W.; Du, Y. C.; Ye, P. D., The Effect of Dielectric Capping on Few-Layer Phosphorene Transistors: Tuning the Schottky Barrier Heights. *IEEE Electron Device Lett.* **2014,** *35* (7), 795-797.
5. Avsar, A.; Vera-Marun, I. J.; Tan, J. Y.; Watanabe, K.; Taniguchi, T.; Neto, A. H. C.; Ozyilmaz, B., Air-Stable Transport in Graphene-Contacted, Fully Encapsulated Ultrathin Black Phosphorus-Based Field-Effect Transistors. *ACS Nano* **2015,** *9* (4), 4138-4145.
